# Differential Phytotoxic Impact of Plant Mediated Silver Nanoparticles (AgNPs) and Silver Nitrate (AgNO_3_) on *Brassica* sp.

**DOI:** 10.3389/fpls.2017.01501

**Published:** 2017-10-12

**Authors:** Kanchan Vishwakarma, Shweta, Neha Upadhyay, Jaspreet Singh, Shiliang Liu, Vijay P. Singh, Sheo M. Prasad, Devendra K. Chauhan, Durgesh K. Tripathi, Shivesh Sharma

**Affiliations:** ^1^Department of Biotechnology, Motilal Nehru National Institute of Technology Allahabad, Allahabad, India; ^2^D D Pant Interdisciplinary Research Lab, Department of Botany, University of Allahabad, Allahabad, India; ^3^College of Landscape Architecture, Sichuan Agricultural University, Chengdu, China; ^4^College of Agriculture, Food and Natural Resources, University of Missouri, Columbia, MO, United States; ^5^Government Ramanuj Pratap Singhdev Post Graduate College, Baikunthpur, India; ^6^Ranjan Plant Physiology and Biochemistry Laboratory, Department of Botany, University of Allahabad, Allahabad, India; ^7^Centre for Medical Diagnostic and Research, Motilal Nehru National Institute of Technology Allahabad, Allahabad, India

**Keywords:** AgNPs, AgNO_3_, plant growth, *Brassica*, photosynthetic parameters

## Abstract

Continuous formation and utilization of nanoparticles (NPs) have resulted into significant discharge of nanosized particles into the environment. NPs find applications in numerous products and agriculture sector, and gaining importance in recent years. In the present study, silver nanoparticles (AgNPs) were biosynthesized from silver nitrate (AgNO_3_) by green synthesis approach using *Aloe vera* extract. Mustard (*Brassica* sp.) seedlings were grown hydroponically and toxicity of both AgNP and AgNO_3_ (as ionic Ag^+^) was assessed at various concentrations (1 and 3 mM) by analyzing shoot and root length, fresh mass, protein content, photosynthetic pigments and performance, cell viability, oxidative damage, DNA degradation and enzyme activities. The results revealed that both AgNPs and AgNO_3_ declined growth of *Brassica* seedlings due to enhanced accumulation of AgNPs and AgNO_3_ that subsequently caused severe inhibition in photosynthesis. Further, the results showed that both AgNPs and AgNO_3_ induced oxidative stress as indicated by histochemical staining of superoxide radical and hydrogen peroxide that was manifested in terms of DNA degradation and cell death. Activities of antioxidants, i.e., ascorbate peroxidase (APX) and catalase (CAT) were inhibited by AgNPs and AgNO_3._ Interestingly, damaging impact of AgNPs was lesser than AgNO_3_ on *Brassica* seedlings which was due to lesser accumulation of AgNPs and better activities of APX and CAT, which resulted in lesser oxidative stress, DNA degradation and cell death. The results of the present study showed differential impact of AgNPs and AgNO_3_ on *Brassica* seedlings, their mode of action, and reasons for their differential impact. The results of the present study could be implied in toxicological research for designing strategies to reduce adverse impact of AgNPs and AgNO_3_ on crop plants.

## Introduction

Nanotechnology deals with the manipulation in material(s) at atomic and molecular level upto the dimension of 100 nanometers. Extensive research on effect of nanoparticles (NPs) on living systems including plants and other organisms has been carried out in last few decades. NPs, due to specific physicochemical properties, are widely being used in day to day life like in medical science, agriculture, environment and other areas under science and technology (Nigam et al., 2009; Chaloupka et al., 2010; Tripathi et al., 2012; Austin et al., 2014; Majdalawieh et al., 2014; Siripattanakul-Ratpukdi and Fürhacker, 2014). NPs show significant changes in physical and chemical characteristics in comparison to their bulk counterparts, which are due to the presence of free dangling bonds and large surface area (Daniel and Astruc, 2004). Although the potential of nanotechnology is widely recognized, however, its optimized use in agriculture to increase crop yield is still debated. Ions from the industrial discharge form cluster after reduction to form NPs, which might be absorbed by plants (Almutairi and Alharbi, 2015). Regulation of level of industrial impurity in water and soil to prevent their adverse effect on vegetation is still a great challenge (Mueller and Nowack, 2008).

The interaction between plants and nanomaterials is still not studied well (Mehta et al., 2016). Reported studies are contradicting in terms of biotransformation, translocation, toxicity, accumulation and absorption of nanomaterials in plants (Husen and Siddiqi, 2014). Investigation on the effect of silver nanoparticles (AgNPs) on plants is still going on (Kaegi et al., 2010; Nowack, 2010). The effect of AgNPs on plants at higher level seems to be dependent on the age and plants species, NP size and its concentration, various experimental conditions and the method and duration of experimental exposure. Studies have shown that AgNPs shed ionic silver into surrounding and cause oxidative stress and inhibition of respiratory enzymes by generating reactive oxygen species (ROS) (Cheng et al., 2010; Tripathi A. et al., 2017). By reviewing various studies, it was perceived that the reason of toxic effect of AgNPs is still not clear, whether it is caused by ionic sliver or it is caused by its intrinsic property. Current literature on the effect of AgNPs on plants is very limited and thus, it becomes important to check their effect, particularly when their constructive and damaging impacts on environment are unknown.

However, it has been shown that as the amount of AgNPs rises in plants, there is a decrement in root length and biomass, which indicates intensification in toxicity (Yin et al., 2011). Lin and Xing (2007) also showed that nanomaterial interaction with plant had variable effect on seed germination, root initiation and growth and it depends upon the properties and concentration of NPs, and species of the plant. AgNP bioaccumulation in plant cells has been shown to be directly dependent on the system reduction potential (Haverkamp and Marshall, 2009). Thus, there are both positive and negative effects of nanomaterials on seed germination and root growth (Krishnaraj et al., 2012). Besides nanomaterials, the impact of AgNO_3_ (precursor of AgNPs) is also very little explored in various plants such as *Cucumis sativus* (Krishnaraj et al., 2012; Tripathi A. et al., 2017). In addition, there are only few studies that have reported the effect of these NPs on mustard plant (Pandey et al., 2014); however, they does not include their effect on cell death and cell morphology.

Therefore, the present study focuses on assessing biochemical changes caused by both silver nitrate (AgNO_3_) and biosynthesized AgNPs at different concentrations on *Brassica* sp. by analyzing seedlings growth, accumulation, oxidative stress, DNA degradation and cell viability and activities of ascorbate peroxidase and catalase.

## Materials and Methods

### Synthesis of Silver Nanoparticles Using Green Synthesis Approach

*Aloe vera* leaves that seem to be healthy were taken from Roxburgh Botanical Garden, University of Allahabad, India. Sterilized plastic bags were taken and leaves were stored in it. Twenty gram leaves were taken, subjected to washing with double distilled water, dried and chopped into fine pieces to prepare leaf extract. A glass beaker was used to boil the finely cut leaves in 100 ml de-ionized water for 20 min. The content obtained after boiling was subjected to filtration by using Whatman No.1 filter paper. To optimize the synthesis of AgNPs, its precursor (AgNO_3_) at different concentrations (40, 60, 80, 100, and 120 mM) was freshly prepared in de-ionized water. To 90 ml of AgNO_3_ solution, slow addition of 10 ml of leaf extract was performed with continuous stirring for 20 min followed by incubation for 24 h at room temperature (RT) with sample analysis at every 4 h. Initially, the colorless solution was turned into pale yellow color and after 24 h, the color was altered from pale yellow to reddish brown indicating formation of AgNPs. The solution was then subjected to centrifugation at 12,000 rpm for 15 min, and dispersed in de-ionized water to remove any other biological material with constant stirring at 50–60°C followed by three washings. Spectrum scan was taken on UV-Visible Spectrophotometer from 360 to 700 nm. After optimization, NPs so obtained were dried to get a fine powder. Estimation of Ag was performed as per method described in Tripathi D.K. et al. (2017).

### Characterization of Silver Nanoparticles

Ultraviolet-visible spectrophotometer (Eppendorf BioSpectro-meter) was used to characterize AgNPs. Scanning was done in the range of 300–700 nm for the samples by taking de-ionized water as a blank. The shape and size of AgNPs were further determined by transmission electron microscopy (TEM).

### Plant Material and Culture Conditions

Mustard (*Brassica* sp.) seeds were procured from the certified supplier of the Allahabad district. Surface sterilization for uniform-sized seeds was performed by using 10% (v*/*v) sodium hypochlorite solution for 10 min. Seeds were then washed with double-distilled water and soaked for 12 h to break dormancy. On next day, seeds were wrapped in cotton cloth to allow germination. Healthy looking uniform-sized seeds were kept in Petri plates (150 mm, Riviera^TM^) lined with Whatman No. 1 having half strength Hoagland solution followed by subsequent germination at 25 ± 2°C for 4 days in the dark. After germination, five uniform length seedlings were placed in 40 ml half strength Hoagland solution per pot and subjected to the AgNP and AgNO_3_ treatments at concentrations of 1 and 3 mM along with control seedlings.

### Estimation of Growth

To estimate growth, 10 seedlings from control and treated samples were selected randomly and then weighed for fresh mass estimation. Length of root and shoot was determined by using centimeter scale. Fresh mass of root and shoot of treated and untreated *Brassica* seedlings was measured as described by Dwivedi et al. (2015).

### Oxidative Stress

Histochemical staining of superoxide radical (O_2_^•–^) was performed according to the method of Kariola et al. (2006). Root sections were kept in petri dishes followed by addition of 6 mM nitro blue tetrazolium (NBT) solution along with 50 mM sodium phosphate (pH: 7.5) and 10 mM sodium azide in the dark for 12 h. Further, root samples were soaked in lactate-glycerol-ethanol (1:1:4 v/v) to stop the O_2_^•–^ reaction, followed by subsequent boiling in water for 5 min. Fifty percent ethanol was used to preserve the cleared roots which was further utilized for photography. The protocol of Thordal-Christensen et al. (1997) was utilized to histochemically stain the roots for the presence of hydrogen peroxide (H_2_O_2_) using 3, 30-diaminobenzidine (DAB) staining. In brief, roots were washed with double distilled water then kept in 1% DAB (pH 3.8; Sigma, United States) for 8 h at 25°C in the light. After staining, samples were subjected to washing and immediately followed by submerging and boiling in 95% ethanol two times for 10 min. Slides were prepared and photographed.

### Biochemical Analyses

Catalase (CAT: EC 1.11.3.6) activity was determined in terms of reduction in optical density due to dissociation of H_2_O_2_ molecules which was recorded at 240 nm having an extinction coefficient of 39.4 mM^-1^ cm^-1^ (Aebi, 1984). A unit of enzyme activity was considered in terms of 1 nmol H_2_O_2_ dissociated min^-1^. Ascorbate peroxidase (APX: EC 1.11.1.11) activity was measured as per the protocol of Nakano and Asada (1981). A unit of enzyme activity was considered in terms of 1 nmol ascorbate oxidized min^-1^. Protein in each sample was determined as per method of Bradford (1976).

### Measurement of Total Chlorophyll and Chlorophyll *a* Fluorescence

To determine total chlorophyll, 20 mg fresh leaves were taken from control and treated seedlings. Leaves were then subjected to crushing in 80% acetone followed by pigment extraction and centrifugation. Absorbance of the extract was taken at 663 and 646 nm by using Eppendorf UV-VIS Spectrophotometer. Total chlorophyll was measured according to the method of Lichtenthaler (1987). For measurement of photosynthetic performance of seedlings, chlorophyll *a* fluorescence was observed in the dark-adapted (30 min) state of leaves with the help of hand-held leaf fluorometer (Fluor Pen FP 100, Photon System Instrument, Czech Republic). The fluorescence parameters including maximum photochemical efficiency of PS II (F_v_/F_m_), photochemical quenching (qP) and non-photochemical quenching (NPQ) were then measured.

### Plant DNA Extraction and Agarose Gel Electrophoresis

0.5 g plant tissue was taken on clean glass slide and chopped into a paste using clean single edge razor blade. The paste of plant tissue was used to extract DNA by the protocol suggested by Keb-Llanes et al. (2002). The pellet obtained after isolation procedure was suspended in 50 μL Tris-EDTA (TE) buffer. 1% (w/v) agarose gel was made in 1X Tris-acetate-EDTA (TAE) buffer followed by addition of 3 μL ethidium bromide. 5 μL of extracted DNA from each sample along with 1 μL DNA loading dye was loaded into the wells. Gel was run for 30 min at 85 V and visualized for degradation of DNA in the GelDoc (UVITEC Cambridge).

### Measurement of Cell Viability by Fluorescence Spectroscopy and Flow Cytometry

Plant samples were prepared by using the method of Pozarowski and Darzynkiewicz (2004) after slight modifications. Cells (10^6^) were suspended in 0.5 mL phosphate buffer saline (PBS) and fixed in 70% ethanol followed by incubation at 4°C for 30 min. Further, cells were washed in 1X PBS and centrifuged at 1200 rpm for 15 min. Supernatant was removed carefully and the pellet having cells was resuspended in 200 μL (Pozarowski and Darzynkiewicz, 2004).

Plant extract prepared above was subjected to staining with propidium iodide (PI which stain nucleic acids), and analyzed through fluorescence microscopy and flow cytometry (FCM). For fluorescence microscopy, addition of 2 μl PI (50 μg/ml) to 200 μl of plant extract was carried out and subjected to incubation for 20 min in the dark to allow cells to retain the stain. After incubation, cells were taken out from the dark for analysis by fluorescence microscopy. The fluorescence data indicates the basic outline with respect to cells’ relative number and morphology that is based on cells’ auto fluorescence and/or labeling with fluorescent dyes, further helping in characterization of plant cells, resolving them from electronic noise and debris, as well as indicating the cell viability and vitality.

Flow cytometry study was performed using a BD Accuri^TM^ C6 Flow Cytometer having a red laser of 14.7 mW output with 640 nm excitation wavelength and a blue laser of 20 mW output having 488 nm excitation wavelength. For flow cytometry, the cell suspension was stained with 10 μL PI followed by 20 min incubation in dark followed by its analysis in FCM. FCM instrument is based on the detection of forward scattering (FSC) and side scattering (SSC) of light by four different types of fluorescence detectors with optical filters. In the present work, red color fluorescence detector (FL2, 585 nm; PE/PI) was utilized.

### Statistical Analysis

All values are means of three independent experiments. The significance of data was confirmed by applying one-way analysis of variance (ANOVA). Comparison with the control and treatment’s means was carried out by applying Duncan’s Multiple Range Test (DMRT) at *p* < 0.05 significance level.

## Results

### Synthesis of Nanoparticles by Green Synthesis Process

AgNPs were synthesized in the reaction mixture containing 120 mM AgNO_3_ and a visible change in color from colorless to brown was observed within 24 h. Supplementary Figures S1a,b shows scanning spectra of concentration and time optimization and Supplementary Figures S2a,b shows difference in color of AgNO_3_ and AgNPs after synthesis.

### Characterization of Synthesized Nanoparticles

**FIGURE 1:**
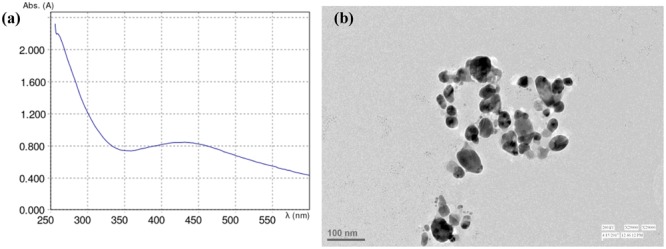
**(a)** Absorption spectrum and **(b)** TEM image of AgNPs formed in the reaction media.

The absorbance maxima of formed AgNPs were noticed at 425 nm. The peak was observed in the range of 420–500 nm by UV-Visible spectrophotometer (**Figure 1a**). The TEM image of AgNPs synthesized by using plant extract confirmed the reduction of silver by *Aloe vera* and showed an average size of 47 nm for AgNPs (**Figure 1b**).

### Effect on Growth Parameters

**FIGURE 2:**
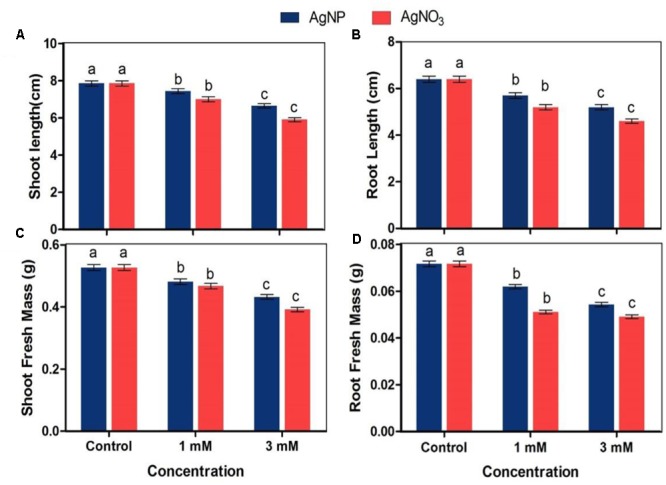
Impact of various concentrations of AgNPs and AgNO_3_ on **(A)** shoot length, **(B)** root length, **(C)** shoot fresh mass, and **(D)** root fresh mass. Data are average ± standard errors of experiments performed in triplicate. Bars followed by different letter(s) show significant difference at *P* < 0.05 significance level according to the DMRT.

Root and shoot fresh mass and length were used to evaluate the impact of AgNO_3_ and AgNPs on growth of *Brassica* seedlings. The results of root and shoot length clearly revealed that AgNO_3_ treatments (1 and 3 mM) significantly reduced the length with increasing concentration whereas AgNPs showed slight decrease when both were compared with the control. AgNO_3_ at 1 mM concentration showed 10.8 and 18.7% reduction in shoot and root length while AgNP at same concentration observed to cause reduction by only 5.3 and 10.9% for shoot and root, respectively (**Figure 2**). However, significant effect was recorded at higher concentration, i.e., 3 mM AgNO_3_ with 24.8 and 28.1% reduction in shoot and root length as compared to its counterpart AgNPs that showed reduction only by 15.4 and 18.7% for shoot and root, respectively and thus, lesser than AgNO_3_.

The fresh mass of shoot and root of seedlings treated with AgNO_3_ and AgNPs was lower than seedlings grown under normal conditions (control) (**Figure 2**). 1 mM AgNO_3_ caused reduction in shoot and root fresh mass by 11.4 and 28.7% respectively while same concentration of AgNPs led to a reduction by only 8.7 and 13% in shoot and root, respectively. The reduction percentage increased with increasing concentration as there was 18 and 24.2% reduction in shoot and root with 3 mM AgNPs, while it was 25.6 and 31.5%, respectively with 3 mM AgNO_3_. The observed results indicate that AgNO_3_ caused more harmful impact on growth characteristics of plant than the biosynthesized AgNPs.

### Effect on Photosynthetic Pigments and Photosynthetic Performance

**FIGURE 3:**
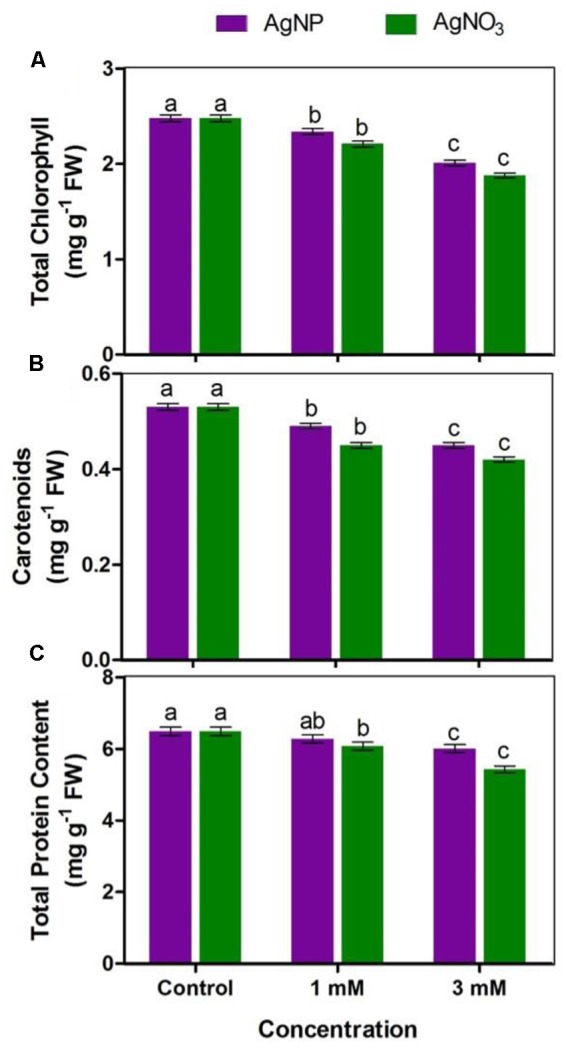
Impact of various concentrations of AgNPs and AgNO_3_ on **(A)** total chlorophyll, **(B)** carotenoids, and **(C)** total protein content. Data are average ± standard errors of experiments performed in triplicate. Bars followed by different letter(s) show significant difference at *P* < 0.05 significance level according to the DMRT.

AgNO_3_ and AgNP treatments (1 and 3 mM) decreased total chlorophyll and carotenoids concentration while the impact of AgNPs at respective concentrations was lesser as compared to the control (**Figures 3A,B**). However, it was clear that percentage reduction for carotenoids was lesser than chlorophylls.

**FIGURE 4:**
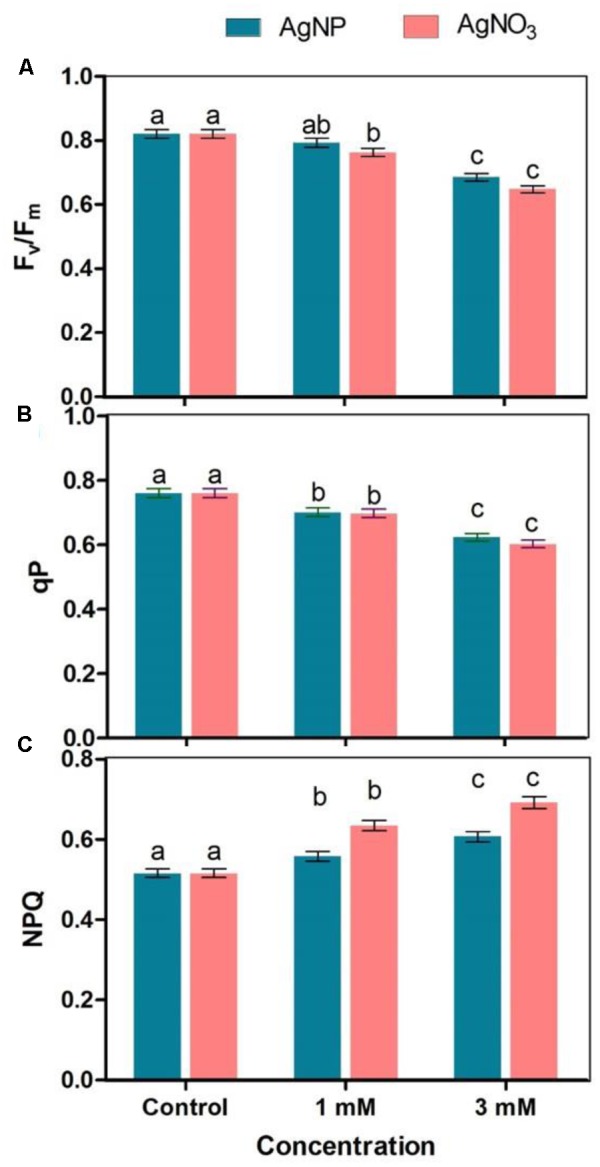
Effect of different concentrations of AgNPs and AgNO_3_ on photosynthetic parameters such as **(A)** F_v_/F_m_, **(B)** qP: photochemical quenching, and **(C)** NPQ, non-photochemical quenching. Data are average ± standard errors of experiments performed in triplicate. Bars followed by different letter(s) show significant difference at *P* < 0.05 significance level according to the DMRT.

A vision about the health of photosynthetic systems in the leaves is provided by chlorophyll *a* fluorescence. The results showed that reduction in F_v_/F_m_ was significant when plants were treated with AgNO_3_ as compared to the control (**Figure 4A**). The effect of AgNO_3_ (1 and 3 mM) was greater than the effect of AgNPs in *Brassica* seedlings. Photochemical (qP) and non-photochemical quenching (NPQ) were also tested under stressed conditions (**Figures 4B,C**). Under given treatments, decrease in qP with increasing values of NPQ were observed in comparison to the control (**Figure 4C**). However, there were no significant changes in NPQ among AgNPs and AgNO_3_ treatments.

### Protein Content

Total protein content in *Brassica* treated with AgNO_3_ and AgNPs was found to be lesser than protein level in control *Brassica* seedlings (**Figure 3C**). 3 mM of AgNO_3_ showed greater reduction (16%) with respect to AgNPs at same concentration (7%), when both were compared with the control. In addition, at 1 mM concentration of AgNPs and AgNO_3_, reduction in total protein content was observed (i.e., 3 and 6%, respectively). The data clearly indicate that damaging effect of AgNO_3_ was more than AgNPs.

### Enzyme Activities

**FIGURE 5:**
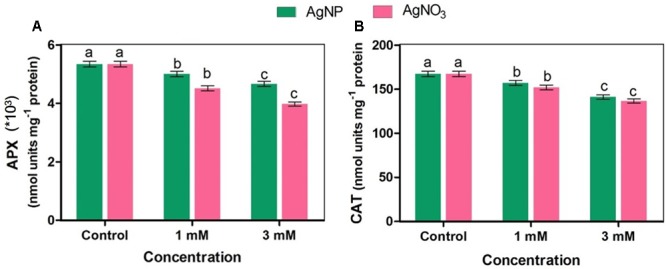
Effect of different concentrations of AgNPs and AgNO_3_ on enzymes **(A)** APX: ascorbate peroxidase, and **(B)** CAT, catalase. Data are average ± standard errors of experiments performed in triplicate. Bars followed by different letter(s) show significant difference at *P* < 0.05 significance level according to the DMRT.

Ascorbate peroxidase (APX) enzyme is involved in dissociation of H_2_O_2_ into water and oxygen by utilizing ascorbate as a particular electron donor, while catalase (CAT) also dissociates H_2_O_2_ into water and oxygen without any external use of reductant(s). The activities of APX and CAT enzymes were significantly inhibited when *Brassica* seedlings were treated with AgNO_3_ while inhibition in their activities in the presence of AgNP was found to be lesser than AgNO_3_ as compared to the control (**Figure 5**).

### Accumulation

**FIGURE 6:**
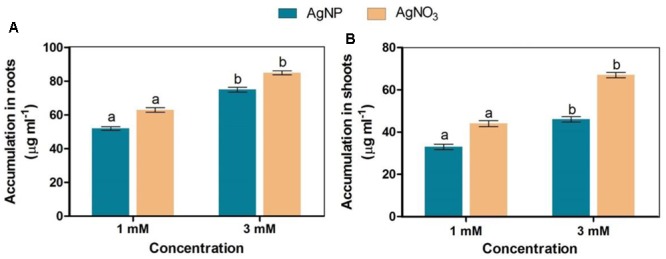
Accumulation status of AgNPs and AgNO_3_ in **(A)** roots and **(B)** shoots. Data are average ± standard errors of experiments performed in triplicate. Bars followed by different letter(s) show significant difference at *P* < 0.05 significance level according to the DMRT.

*Brassica* seedlings exposed to AgNO_3_ and AgNPs showed their higher accumulation in roots than shoots. However, it was noticed that AgNO_3_ at 1 and 3 mM accumulated more in both roots and shoots than AgNPs (**Figure 6**).

### Oxidative Damage

**FIGURE 7:**
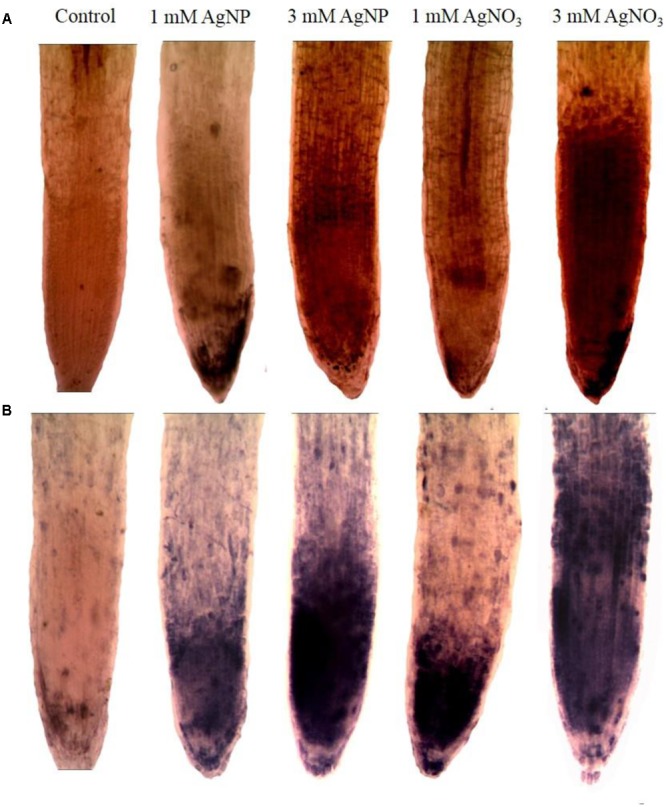
Impact of AgNPs and AgNO_3_ on root oxidative stress after staining with **(A)** DAB and **(B)** NBT.

The results showed that AgNO_3_ enhanced accumulation of superoxide radical and hydrogen peroxide in *Brassica* more than AgNPs as indicated by histochemical staining of roots by NBT (for superoxide radical) and DAB (for H_2_O_2_) dye reduction tests. In addition, the appearance of root hairs were reduced under AgNO_3_ treated seedlings (**Figure 7**).

### Degradation of DNA

**FIGURE 8:**
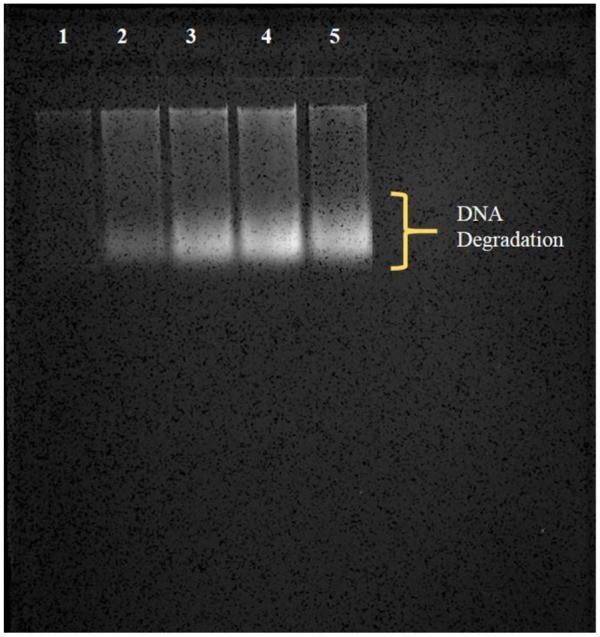
Observation of degradation of DNA (in the form of smear) by agarose gel electrophoresis; (1) control, (2) 1 mM AgNP, (3) 1 mM AgNO_3_, (4) 3 mM AgNP, (5) 3 mM AgNO_3_.

DNA of the *Brassica* seedlings treated with AgNPs and AgNO_3_ was isolated by CTAB method followed by its suspension in TE buffer and stored at -40°C till further use. Thereafter, the extracted and stored DNA of each sample was run in agarose gel and it was found that a degradation of DNA took place in samples treated with AgNP and AgNO_3_ in comparison to the control (**Figure 8**). However, more degradation was observed at 3 mM AgNO_3_ as well as 3 mM AgNPs concentration when compared to control where no degradation was observed.

### Cell Viability Assessment with Fluorescence Microscopy

**FIGURE 9:**
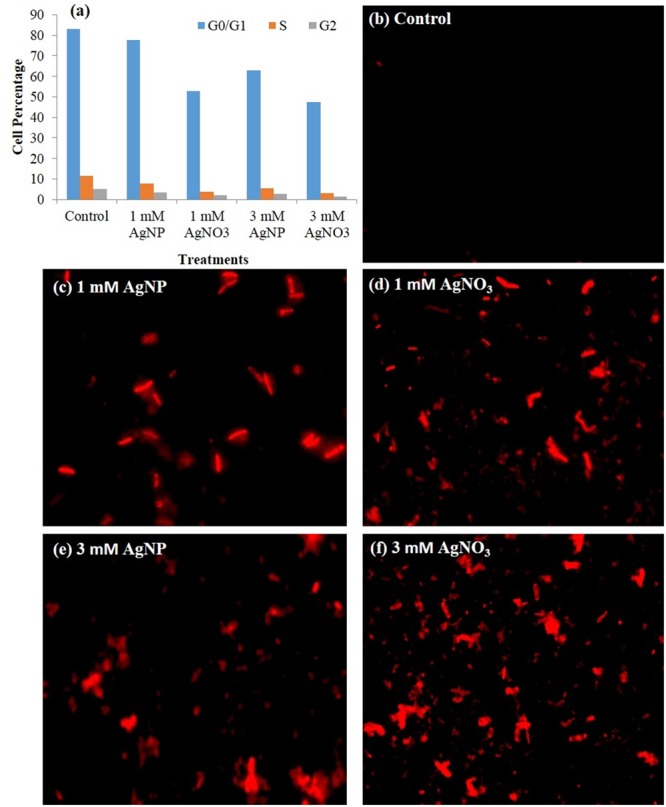
**(a)** Cell cycle profile of plant analyzed by flow cytometry showing percent of cells in each phase, **(b–f)** Fluorescence microscopy images of plant cells treated with different concentrations of AgNP and AgNO_3_, and stained with PI.

*Brassica* seedlings treated with different concentrations of AgNPs and AgNO_3_ were taken and plant extract was prepared. Qualitative assessment of cell viability was done using fluorescence microscopy after staining the cells in the extract with PI. PI is considered as a marker of the cell death because of its exclusion by cell membrane of live cells. Hence, the fluorescence conferred by the dye generally indicates the presence of cells in which cell membrane is damaged or ruptured. After staining, dead cells take up the dye and appear as a red spots upon observation by fluorescence microscopy (**Figures 9b–f**). It is evident from the results that cells under AgNO_3_ treatment showed more cell death (more stained cells) in comparison to the AgNPs. Number of cell death was higher in treated seedlings than the control (**Figures 9b–f**).

### Cell Cycle Analysis by Flow Cytometry

In this study, extract of treated seedlings was stained with PI and observed by flow cytometry. Cells gave high PI fluorescence intensity that indicates the percent cell death in the presence of AgNO_3_ and AgNPs (**Figure 9a**). The flow cytometric bar graph showed dominant G_0_/G_1_ profile. It was evident from the results that cell cycle dynamics of various populations was influenced by treatments given. The plants exposed to 3 mM AgNO_3_ and AgNP showed a decline in subpopulation of cells in G_0_/G_1_, S, G_2_ phases (**Figure 9a**).

## Discussion

The present study was undertaken to investigate the differential impact of AgNO_3_ and AgNPs on mustard (*Brassica* sp.) seedlings. AgNO_3_ and AgNPs exposure significantly reduced growth parameters in mustard seedlings that might be directly linked with reduction in photosynthetic performance.

More reduction in fresh mass and length of root and shoot, photosynthetic parameters, and protein content was observed under AgNO_3_ treatment than that under AgNPs when impacts of both were compared with the control. Similarly, in a study by Krishnaraj et al. (2012) showed that plants when given AgNPs exhibited no severe toxic impacts on morphology studied by the scanning electron microscopy (SEM). However, growth retarded at high concentrations of AgNO_3_ treatment. It was previously stated that reduction in growth characteristics under AgNO_3_ is due to increased uptake of silver by plants (Harris and Bali, 2008). The above observations suggest that Ag in both forms, i.e., bulk silver and nano silver might show interaction with proteins and components of the plant system. There are only few reports that showed interaction of NPs with plant systems, but observations are mixed in nature (positive or negative). In a study, the impact of AgNPs on growth parameters of three plant species, i.e., watermelon, corn, and zucchini were examined and it was noticed that their germination rates enhanced in response to AgNPs (Almutairi and Alharbi, 2015). Although, AgNPs imparted toxicity on corn root elongation, however, growth of watermelon and zucchini seedlings was positively influenced by AgNPs (Almutairi and Alharbi, 2015). Additionally, in the present study, some evident signs such as alterations in shoots and root growth were also observed (**Figure 2**). It might be because of uptake of AgNPs through seeds and changes in membrane and other cell structures, as well as defensive mechanisms (Blaser et al., 2008), and modification in cell division and/or cell elongation (Singh et al., 2014). The result that AgNO_3_ caused more deleterious effects on growth of seedling than AgNPs was in agreement with the work carried out by Sarabi et al. (2015). Interestingly, in this research, there was no definite effect of high concentration of AgNP on mustard seeds. Also, the observed effects of AgNO_3_ and AgNPs on root and shoot length were in accordance with the study by Pandey et al. (2014).

In our study, a minor decline in length of shoot and root of seedlings under AgNPs treatment was observed (**Figures 2A,B**). However, few other studies have noticed that association of Ag^+^ ions with roots distorted its epidermis structure and changed anatomical properties of sunflower plant (Krizkova et al., 2008). Furthermore, the same plant was observed, and arrested growth of various parts of plant along with damaged root hairs were noticed (Krizkova et al., 2008), while treatment with alumina (Yang and Watts, 2005) and copper (Lee et al., 2008) NPs have retarded the growth of seedlings. Interestingly, not much harmful impact like root hair distortion, and changes in root morphology were observed in the roots of AgNP treated plants, while plants that were given AgNO_3_ showed more accumulation in roots, which retarded growth of plant (**Figures 2, 6**). This observation is in accordance with earlier studies which mentioned that maximum silver present in the plant remains associated within the roots and the translocation factor ([Ag]_shoot_/[Ag]_root_) is very low (Yin et al., 2012; Pandey et al., 2014).

Photosynthetic efficiency can be detected by measuring some parameters of chlorophyll *a* fluorescence *in vivo* under normal and stressed conditions (Pandey et al., 2014). The results confirmed that under AgNO_3_ exposure, F_v_/F_m_ (a reliable marker of photosynthetic efficiency) and qP were markedly declined (**Figures 4A,B**), which could be correlated with reduction in total chlorophyll (**Figure 3A**). However, these parameters when tested under AgNPs exposure showed lesser reduction when compared to AgNO_3_ treatment, but were significant when compared to the control (**Figure 4**). However, enhanced quantum efficiency and more chlorophyll content were measured in mustard leaves treated with AgNPs (Sharma et al., 2012). Earlier studies have shown that the activity of photosystem II (PS II) is influenced by various stresses that resulted in decreased F_v_/F_m_ and qP (Xing et al., 2010; Singh et al., 2014). Substantial decrease in F_v_/F_m_ (**Figure 4A**) and qP (**Figure 4B**) gives the sign of modifications in structure and function of photosynthetic process which might be linked with decrease in biomass accumulation in mustard seedlings. Furthermore, Genty et al. (1990) observed that rise in the values of NPQ when subjected to stressed situations might be due to the down-regulation of photosystem II to evade additional reduction of Q_A_ so as to equalize the decreased need for electrons through NADPH. In this study, NPQ was enhanced significantly under AgNO_3_ treatment while its enhancement was lesser under AgNPs treatment, suggesting that AgNPs allow the proper working of electron transport chain than AgNO_3_ (**Figure 4C**).

The decrease in growth of mustard seedlings subjected to AgNO_3_ could be correlated with generation of more ROS as measured by histochemical staining by NBT and DAB (**Figure 7**), which are responsible for causing lipid peroxidation and protein oxidation. AgNPs treatment caused little effect on oxidative stress as compared to AgNO_3_. The reason of such remarkable reduction in growth after treatments might be attributed to ROS mediated damages to the biomolecules linked with the photosynthetic apparatus, which mainly involved the membranes of energy transduction system as reported in a recent study (Tripathi D.K. et al., 2017). Earlier, it was studied that stress factor accelerates the generation of ROS (H_2_O_2_) (Sharma et al., 2012; Jiang et al., 2017). For regulation of ROS levels inside the cell, plants have fine-tuned antioxidant defense system. In the present study, noteworthy (*P* < 0.05) decrease in APX (**Figure 5A**) and CAT (**Figure 5B**) activity was observed in AgNO_3_ treated seedlings, as compared to control and AgNPs. Other NPs like CuNP and its bulk CuCl_2_ have also been found to decrease CAT activity because of direct involvement of copper to interact with thiol moieties of protein resulting in altered tertiary structure of CAT and ultimate inhibition (Atli et al., 2006). Possibly a similar mechanism may operate for Ag resulting in increased production of ROS. In a study by Sharma et al. (2012), it was shown that AgNPs support the growth of *Brassica juncea* seedlings by controlling their antioxidant level. Similarly, Mehta et al. (2016) observed that AgNPs application did not affect growth parameters in wheat seedlings. Likewise, in the present study, though AgNP enhanced the production of ROS but the increase was not significant when compared to the control. It was in support of a recent study, which showed that such effect of AgNPs was related to the blocking of electrons transfer that induces oxidative stress under light (Zou et al., 2016). As observed here that mustard seedlings under AgNPs treatment showed decrease in CAT and APX activities as compared to the control but relatively lesser reduction was observed than AgNO_3_ (**Figure 5**), this might indicate the interaction of AgNPs with proteins present in cytosol and lipid bilayer; thereby, altering the configuration and damaging the antioxidant defense systems (Saptarshi et al., 2013; Hayashi et al., 2013; McShan et al., 2014; Mendes et al., 2015).

From the results, it was apparent that AgNPs synthesized biologically, were polydispersive in nature, decreased ability of penetrating and bioaccumulation, and posed relatively lesser toxic impact than silver nitrate (**Figure 6**). In general, it is mentioned that AgNPs uptake and translocation across the root cells depend upon type and concentration of ions and plant species (Lin and Xing, 2007; Albanese and Chan, 2011; Dos Santos et al., 2011). Since proteins adsorb on the NP surface almost instantly and then NP-protein complex enters the cell (Saptarshi et al., 2013), therefore it might be probable that due to the reduction in total protein content (**Figure 3C**), reduced accumulation of AgNPs was observed with its increasing concentration.

The observation on impact of AgNO_3_ and AgNPs on total protein content is shown in **Figure 3C**. The high concentrations of AgNO_3_ decreased level of total protein in mustard seedlings as compared to AgNPs and control. It is known from earlier investigations that NPs induce conformational changes in proteins when present in high concentrations. AgNO_3_ treatment at high concentration showed 16% reduction in total protein content when compared to the control and same concentration of AgNPs (6% reduction) (**Figure 3C**). It might be due to the reason that AgNP and AgNO_3_ cause discrete modifications in the proteome of root and shoot (Vannini et al., 2013) and interfere with the protein interactions, cell-signaling and DNA transcription (Saptarshi et al., 2013).

The study showed that there was a substantial reduction in number of live plant cells at high concentration of AgNO_3_ when observed under fluorescence microscopy. The notable increase in fluorescence intensity of PI at high concentrations (**Figures 9b–f**) reflects the increase in cell membrane damage. Furthermore, under AgNPs and AgNO_3_ treatments, difference in cell death was witnessed by changing their concentrations using fluorescence microscopy. Maximum cell death in plant samples corresponds to the presence of greater number of cells that have acquired PI, which might be correlated with the decreased growth of mustard plants with AgNO_3_ treatments as compared to the AgNPs and control. In addition, it was evident from **Figure 8** that degradation of DNA had occurred during treatments of AgNO_3_ and AgNPs as a result of loss in membrane integrity due to which PI is able to intercalate with DNA of plant cells because of uptake and accumulation of silver inside the cells. Still, the results could not clarify the rise in PI fluorescence intensity at extreme stress conditions, whether it might be either due to the damage in membrane at localized position or due to the complete loss of membrane integrity. Treatment of AgNO_3_ and AgNPs on plant cells influenced the membrane potential (difference in internal and external electrical potential of a cell) of cytoplasm resulting in damaged membrane permeability. In control, very less intensity was observed, whereas a notable increase was observed at 3 mM AgNO_3_ concentration. However, at 3 mM AgNPs treatment, the intensity was found to be less in comparison to the 3 mM AgNO_3_ concentration (**Figures 9e,f**). Cell cycle dynamics studied by flow cytometry was also found to be affected under various treatments. Variations in the cell percentage occurred at different phases; decreasing the number of cells in S, G_2_ phase and G_0_/G_1_ phase indicating that synthesis of DNA was hampered during cell cycle under treatments. (**Figure 9a**). It was also supported by the result of gel electrophoresis that disruption of nucleic acids occurred in treatments; thereby resulting in degradation of DNA (**Figure 8**). Therefore, from the results, it is concluded that the toxicity of AgNO_3_ was more prominent than AgNPs.

## Conclusion

It is evident from the results that AgNO_3_ posed more deleterious effects on growth of mustard seedling. Although, silver NPs did affect the growth at high concentrations, its impact is lower than that of AgNO_3_. Hence, from this study, it can be concluded that the interacting ability of silver prompted the stressed condition for growth and metabolism of *Brassica* sp. Among the two treatment conditions, AgNO_3_ treated plants had lower levels of total protein, carotenoids, CAT and APX activities while a somewhat higher level was observed in AgNPs treated seedlings. The cell viability assessment by fluorescence microscopy and cell cycle analysis by flow cytometry concluded that treatment with AgNO_3_ posed harmful effects on structural and functional properties of the cell. Therefore, in view of present findings, AgNO_3_ interaction retards the growth of plants by imparting toxicity, whereas biologically synthesized AgNPs interaction on the growth and metabolism of mustard seedlings impose mild stress condition.

## Author Contributions

KV, DT, S, VS, SP, and NU designed the manuscript. KV, DT, S, NU, and JS performed the experiment and wrote the manuscript. SL, DT, DC, and SS critically evaluated the manuscript.

## Conflict of Interest Statement

The authors declare that the research was conducted in the absence of any commercial or financial relationships that could be construed as a potential conflict of interest.

## References

[B1] Aebi I. I. (1984). Catalase *in vitro*. *Methods Enzymol.* 105 121–126. 10.1016/S0076-6879(84)05016-3 6727660

[B2] Albanese A. Chan W. C. W. (2011). Effect of gold nanoparticle aggregation on cell uptake and toxicity. *ACS Nano* 5 5478–5489. 10.1021/nn2007496 21692495

[B3] Almutairi Z. M. Alharbi A. (2015). Effect of silver nanoparticles on seed germination of crop plants. *J. Adv. Agric.* 4 283–288. 10.1016/j.scitotenv.2013.02.059 23532040

[B4] Atli G. Alptekin Ö Tükel S. Canli M. (2006). Response of catalase activity to Ag^+^, Cd^2+^, Cr^6+^, Cu^2+^ and Zn^2+^ in five tissues of freshwater fish *Oreochromis niloticus*. *Comp. Biochem. Phys. C. Toxicol Pharmacol.* 143 218–224. 10.1016/j.cbpc.2006.02.003 16581305

[B5] Austin L. A. Mackey M. A. Dreaden E. C. El-Sayed M. A. (2014). The optical, photothermal, and facile surface chemical properties of gold and silver nanoparticles in biodiagnostics, therapy, and drug delivery. *Arch. Toxicol.* 88 1391–1417. 10.1007/s00204-014-1245-3 24894431 PMC4136654

[B6] Blaser S. A. Scheringer M. Macleod M. Hungerbühler K. (2008). Estimation of cumulative aquatic exposure and risk due to silver: contribution of nano-functionalized plastics and textiles. *Sci. Total. Environ.* 390 396–409. 10.1016/j.scitotenv.2007.10.010 18031795

[B7] Bradford M. M. (1976). A rapid and sensitive method for the quantitation of microgram quantities of protein utilizing the principle of protein-dye binding. *Anal. Biochem.* 72 248–254. 10.1016/0003-2697(76)90527-3 942051

[B8] Chaloupka K. Malam Y. Seifalian A. M. (2010). Nanosilver as a new generation of nanoproduct in biomedical applications. *Trends Biotechnol.* 28 580–588. 10.1016/j.tibtech.2010.07.006 20724010

[B9] Cheng L. Shao M. Zhang M. Ma D. D. (2010). An ultrasensitive method to detect dopamine from single mouse brain cell: surface-enhanced Raman scattering on Ag nanoparticles from beta-silver vanadate and copper. *Sci. Adv. Mater.* 2 386–389. 10.1039/b802405g 18473053

[B10] Daniel M. C. Astruc D. (2004). Gold nanoparticles: assembly, supramolecular chemistry, quantum-size-related properties, and applications toward biology, catalysis, and nanotechnology. *Chem. Rev.* 104 293–346. 10.1021/cr030698+ 14719978

[B11] Dos Santos T. Varela J. Lynch I. Salvati A. Dawson K. A. (2011). Quantitative assessment of the comparative nanoparticle-uptake efficiency of a range of cell lines. *Small* 7 3341–3349. 10.1002/smll.201101076 22009913

[B12] Dwivedi R. Singh V. P. Kumar J. Prasad S. M. (2015). Differential physiological and biochemical responses of two *Vigna* species under enhanced UV-B radiation. *J. Radiat. Res. Appl. Sci.* 8 173–181. 10.1016/j.jrras.2014.12.002

[B13] Genty B. Harbinson J. Briantais J. M. Baker N. R. (1990). The relationship between non-photochemical quenching of chlorophyll fluorescence and the rate of photosystem 2 photochemistry in leaves. *Photosynth. Res.* 25 249–257. 10.1007/BF00033166 24420355

[B14] Harris A. T. Bali R. (2008). On the formation and extent of uptake of silver nanoparticles by live plants. *J. Nanopart. Res.* 10 691–695. 10.1007/s11051-007-9288-5

[B15] Haverkamp R. G. Marshall A. T. (2009). The mechanism of metal nanoparticle formation in plants: limits on accumulation. *J. Nanopart. Res.* 11 1453–1463. 10.1007/s11051-008-9533-6

[B16] Hayashi Y. Miclaus T. Scavenius C. Kwiatkowska K. Sobota A. Engelmann P. (2013). Species differences take shape at nanoparticles: protein corona made of the native repertoire assists cellular interaction. *Environ. Sci. Technol.* 47 14367–14375. 10.1021/es404132w 24245550

[B17] Husen A. Siddiqi K. S. (2014). Phytosynthesis of nanoparticles: concept, controversy and application. *Nanoscale Res. Lett.* 9:229. 10.1186/1556-276X-9-229 24910577 PMC4031915

[B18] Jiang H. S. Yin L. Y. Ren N. N. Zhao S. T. Li Z. Zhi Y. (2017). Silver nanoparticles induced reactive oxygen species via photosynthetic energy transport imbalance in an aquatic plant. *Nanotoxicology* 11 157–167. 10.1080/17435390.2017.1278802 28044463

[B19] Kaegi R. Sinnet B. Zuleeg S. Hagendorfer H. Mueller E. Vonbank R. (2010). Release of silver nanoparticles from outdoor facades. *Environ. Pollut.* 158 2900–2905. 10.1016/j.envpol.2010.06.009 20621404

[B20] Kariola T. Brader G. Helenius E. Li J. Heino P. Palva E. T. (2006). *Early responsive to dehydration 15* a negative regulator of ABA-responses in *Arabidopsis*. *Plant Physiol.* 142 1559–1573. 10.1104/pp.106.086223 17056758 PMC1676049

[B21] Keb-Llanes M. González G. Chi-Manzanero B. Infante D. (2002). A rapid and simple method for small-scale DNA extraction in Agavaceae and other tropical plants. *Plant Mol. Biol. Rep.* 20 299–300. 10.1007/BF02782465

[B22] Krishnaraj C. Jagan E. G. Ramachandran R. Abirami S. M. Mohan N. Kalaichelvan P. T. (2012). Effect of biologically synthesized silver nanoparticles on *Bacopa monnieri* (Linn.) Wettst. plant growth metabolism. *Process. Biochem.* 47 651–658. 10.1016/j.procbio.2012.01.006

[B23] Krizkova S. Ryant P. Krystofova O. Adam V. Galiova M. Beklova M. (2008). Multi-instrumental analysis of tissues of sunflower plants treated with silver(I) ions—plants as bioindicators of environmental pollution. *Sensors* 8 445–463. 10.3390/s8010445 27879716 PMC3681137

[B24] Lee W. M. An Y. J. Yoon H. Kweon H. S. (2008). Toxicity and bioavailability of copper nanoparticles to the terrestrial plants mung bean (*Phaseolus radiatus*) and wheat (*Triticum aestivum*): plant agar test for water-insoluble nanoparticles. *Environ. Toxicol. Chem.* 27 1915–1921. 10.1897/07-481.1 19086317

[B25] Lichtenthaler H. K. (1987). “Chlorophylls and carotenoids, the pigments of photosynthetic biomembranes,” in *Methods in Enzymology* eds Douce R. Packer L. (New York, NY: Academic Press) 350–382.

[B26] Lin D. Xing B. (2007). Phytotoxicity of nanoparticles: inhibition of seed germination and root growth. *Environ. Pollut.* 150 243–250. 10.1016/j.envpol.2007.01.016 17374428

[B27] Majdalawieh A. Kanan M. C. El-Kadri O. Kanan S. M. (2014). Recent advances in gold and silver nanoparticles: synthesis and applications. *J. Nanosci. Nanotechnol.* 14 4757–4780. 10.1166/jnn.2014.9526 24757945

[B28] Mazumdar H. Ahmed G. U. (2011). Synthesis of silver nanoparticles and its adverse effect on seed germinations in *Oryza sativa, Vigna radiate* and *Brassica campestris*. *Int. J. Adv. Biotechnol. Res.* 2 404–413.

[B29] McShan D. Ray P. C. Yu H. (2014). Molecular toxicity mechanism of nanosilver. *J. Food Drug Anal.* 22 116–127. 10.1016/j.jfda.2014.01.010 24673909 PMC4281024

[B30] Mehta C. M. Srivastava R. Arora S. Sharma A. K. (2016). Impact assessment of silver nanoparticles on plant growth and soil bacterial diversity. *3 Biotech* 6 254. 10.1007/s13205-016-0567-7 28330326 PMC5125160

[B31] Mendes L. A. Maria V. L. Scott-Fordsmand J. J. Amorim M. J. (2015). Ag nanoparticles (Ag NM300K) in the terrestrial environment: effects at population and cellular level in *Folsomia candida* (Collembola). *Int. J. Environ. Res. Public Health* 12 12530–12542. 10.3390/ijerph121012530 26473892 PMC4626984

[B32] Mueller N. C. Nowack B. (2008). Exposure modeling of engineered nanoparticles in the environment. *Environ. Sci. Technol.* 42 4447–4453. 10.1021/es7029637 18605569

[B33] Nakano Y. Asada K. (1981). Hydrogen peroxide is scavenged by Ascorbate specific peroxidase in spinach chloroplasts. *Plant Cell Physiol.* 22 867–880.

[B34] Nigam N. Kumar S. Dutta P. K. Ghose T. (2009). Preparation and characterization of chitosan based silver nanocomposites as bio-optical material. *Asian Chitin J.* 5 97–100.

[B35] Nowack B. (2010). Nanosilver revisited downstream. *Science* 330 1054–1055. 10.1126/science.1198074 21097924

[B36] Pandey C. Khan E. Mishra A. Sardar M. Gupta M. (2014). Silver nanoparticles and its effect on seed germination and physiology in *Brassica juncea* L. (Indian mustard) plant. *Adv. Sci. Lett.* 20 1673–1676. 10.1166/asl.2014.5518

[B37] Pozarowski P. Darzynkiewicz Z. (2004). Analysis of cell cycle by flow cytometry. *Methods Mol. Biol.* 281 301–311. 10.1385/1-59259-811-0:301 15220539

[B38] Saptarshi S. R. Duschl A. Lopata A. L. (2013). Interaction of nanoparticles with proteins: relation to bio-reactivity of the nanoparticle. *J. Nanobiotechnol.* 11:26. 10.1186/1477-3155-11-26 23870291 PMC3720198

[B39] Sarabi M. Afshar A. S. Mahmoodzadeh H. (2015). Physiological analysis of silver nanoparticles and AgNO3 effect to *Brassica napus* L. *J. Chem. Health Risks* 5 285–294.

[B40] Sharma P. Bhatt D. Zaidi M. G. H. Saradhi P. P. Khanna P. K. Arora S. (2012). Silver nanoparticle-mediated enhancement in growth and antioxidant status of *Brassica juncea*. *Appl. Biochem. Biotechnol.* 167 2225–2233. 10.1007/s12010-012-9759-8 22692847

[B41] Singh V. P. Kumar J. Singh S. Prasad S. M. (2014). Dimethoate modifies enhanced UV-B effects on growth, photosynthesis and oxidative stress in mung bean (*Vigna radiata* L.) seedlings: implication of salicylic acid. *Pestic. Biochem. Physiol.* 116 13–23. 10.1016/j.pestbp.2014.09.007 25454516

[B42] Siripattanakul-Ratpukdi S. Fürhacker M. (2014). Review: issues of silver nanoparticles in engineered environmental treatment systems. *Water Air Soil Pollut.* 225 1–18. 10.1007/s11270-014-1939-4

[B43] Thordal-Christensen H. Zhang Z. Wei Y. Collinge D. B. (1997). Subcellular localization of H_2_O_2_ in plants. H_2_O_2_ accumulation in papillae and hypersensitive response during the barley-powdery mildew interaction. *Plant J.* 11 1187–1194. 10.1046/j.1365-313X.1997.11061187.x

[B44] Tripathi A. Liu S. Singh P. K. Kumar N. Pandey A. C. Tripathi D. K. (2017). Differential phytotoxic responses of silver nitrate (AgNO_3_) and silver nanoparticle (AgNps) in *Cucumis sativus* L. *Plant Gene.* (in press) 10.1016/j.plgene.2017.07.005

[B45] Tripathi D. K. Singh S. Singh V. P. Prasad S. M. Dubey N. K. Chauhan D. K. (2017). Silicon nanoparticles more effectively alleviated UV-B stress than silicon in wheat (*Triticum aestivum*) seedlings. *Plant Physiol. Biochem.* 110 70–81. 10.1016/j.plaphy.2016.06.026 27470120

[B46] Tripathi D. K. Singh V. P. Kumar D. Chauhan D. K. (2012). Rice seedlings under cadmium stress: effect of silicon on growth, cadmium uptake, oxidative stress, antioxidant capacity and root and leaf structures. *Chem. Ecol.* 28 281–291. 10.1080/02757540.2011.644789

[B47] Vannini C. Domingo G. Onelli E. Prinsi B. Marsoni M. Espen L. (2013). Morphological and proteomic responses of *Eruca sativa* exposed to silver nanoparticles or silver nitrate. *PLoS ONE* 8:68752. 10.1371/journal.pone.0068752 23874747 PMC3715538

[B48] Xing W. Li D. Liu G. (2010). Antioxidative responses of *Elodea nuttallii* (Planch.) H. St. John to short-term iron exposure. *Plant Physiol. Biochem.* 48 873–878. 10.1016/j.plaphy.2010.08.006 20829054

[B49] Yang L. Watts D. J. (2005). Particle surface characteristics may play an important role in phytotoxicity of alumina nanoparticles. *Toxicol. Lett.* 158 122–132. 10.1016/j.toxlet.2005.03.003 16039401

[B50] Yin L. Benjamin P. C. Bonnie M. M. Justin P. W. Emily S. B. (2012). Effects of silver nanoparticle exposure on germination and early growth of eleven wetland plants. *PLoS ONE* 7:47674. 10.1371/journal.pone.0047674 23091638 PMC3473015

[B51] Yin L. Cheng Y. Espinasse B. Colman B. P. Auffan M. Wiesner M. (2011). More than the ions: the effects of silver nanoparticles on *Lolium multiflorum*. *Environ. Sci. Technol.* 45 2360–2367. 10.1021/es103995x 21341685

[B52] Zou X. Li P. Huang Q. Zhang H. (2016). The different response mechanisms of *Wolffia globosa*: light-induced silver nanoparticle toxicity. *Aquat. Toxicol.* 176 97–105. 10.1016/j.aquatox.2016.04.019 27130969

