# Supplementary material for: Differential Phytotoxic Impact of Plant Mediated Silver Nanoparticles (AgNPs) and Silver Nitrate (AgNO3) on Brassica sp
Source: Front Plant Sci. 2017 Oct 12;8:1501. doi: 10.3389/fpls.2017.01501 (PMC5644052; doi:10.3389/fpls.2017.01501)
Supplement: Supplementary file 1 [file Data_Sheet_1.docx]

**Supplementary Figures**

**Figure S1:** (a) Scanning spectra of concentration optimization of AgNO_3_, (b) Scanning spectra of time optimization of synthesis of AgNPs from AgNO_3_

**Figure S2:** (a) 120 mM AgNO_3_ (b) Silver nitrate solution after incubation in the dark for 24 h.

**(a)**

**(b)**

Figure S1


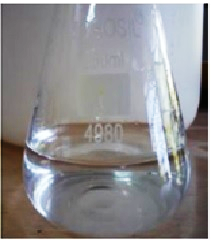

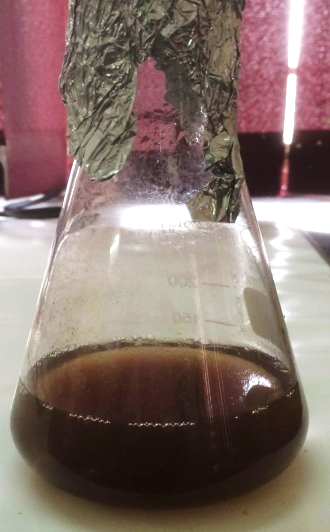


**(b)**

**(a)**

**Figure S2**
